# Algorithm changes in treatment of submandibular gland sialolithiasis

**DOI:** 10.1007/s00405-013-2463-7

**Published:** 2013-04-09

**Authors:** Tomasz Kopeć, Małgorzata Wierzbicka, Witold Szyfter, Małgorzata Leszczyńska

**Affiliations:** ENT Department, Medical University Poznan, Przybyszewski Street 49, 60355 Poznań, Poland

**Keywords:** Chronic sialadenitis, Lithiasis, Sialendoscopy, Open surgery

## Abstract

Our research was conducted to determine the algorithm changes during the treatment of submandibular sialolithiasis. Two time periods were compared between 2004–2008 and 2009–2012. The turning point was December 2008, when sialendoscopy procedure was introduced. In the first period, 48 patients were treated: 31 outpatient duct incisions with stone evacuation and 17 surgical excision of submandibular gland. In the second period, 207 sialendoscopy procedures were performed on 197 patients. Out of this particular group, 158 patients were diagnosed with pathological obstruction of salivary glands and 64 of them were confirmed to have sialolithiasis of submandibular gland. Deposits of calcifications in 40 individuals (62.5 %) affected by sialolithiasis were removed endoscopically; however, in 21 patients, due to the increased circumference of the stone, the intimate association of deposits within the wall of the duct along with its presence inside the deep portions of the gland, double approach (incision of the floor of the mouth in hilar area and sialendoscopy) was performed. Three individuals had their salivary glands totally removed due to the presence of calcified deposits within the glandular parenchyma. Our results allow us to affirm that sialendoscopy is the current treatment of choice for submandibular glands affected by sialoliths. Indication for a complete removal of the gland is becoming uncommon as a first line treatment although still indispensable in chosen cases.

## Introduction

Chronic sialadenitis is one of the major disorders that can cause salivary hypofunction and correct diagnosis and management is essential for its recovery. The classification of this pathological condition has changed in the past decade and nowadays was revised and modified, for new diagnostic (high-resolution ultrasonography, CT and MR sialography and sonoelastography) and therapeutic methods (sialendoscopy) were introduced [12]. Sialolithiasis is the most common cause of inflammatory diseases of large salivary glands and occurs in about 1.2 % of the population [9, 21] mostly in the submandibular gland—87 %. Salivary gland stones are single or multiple, located in the efferent duct distally or proximally, rarely occur intraparenchymally, representing various shapes and sizes. The annual increase in size of salivary stones is estimated at 1 mm [14], and thus the duration of complaints history is crucial for treatment planning.

Contemporary achievement in endoscopy caused strong common belief that stones of up to 4–5 mm in diameter can be successfully removed through sialendoscopy (SE). This applies especially to stones which lie freely in the lumen of the duct and are mobile. In these cases, the stones can be extracted under endoscopic control in more than 80 % of cases [8, 9, 14]. Larger sialoliths may, however, be fragmented in the lumen of the duct, either mechanically or using a laser beam. Lithotripsy (ESWL) is another possibility for the fragmentation of large sialoliths of any size and location; although up to three sessions of lithotripsy may be required. Thus, the introduction of sialendoscopy has significantly reduced the number of submandibular glands removal in the course of sialolithiasis [5, 6, 8, 9, 14, 17]. According to literature data, the use of lithotripsy is effective in 75 % of cases, and in turn, allows for the complete retrieval of stones in half of the cases [2, 3, 6, 7, 22]. The number of successes in the use of lithotripsy clearly decreases with increase in the stone diameter. Despite notable technological progress, 5–10 % of patients with sialolithiasis cannot be successfully treated using minimally invasive techniques [13]. The main cause appears to be the large size of the stones and long-standing history of recurrent inflammations, which lead to the impaction of the sialolith to the wall of the efferent duct. In these cases, the complete removal of the submandibular gland is indispensible. The aim of the study was to analyze the trends in the treatment methods in submandibular sialolithiasis in the past decade, the effectiveness of particular methods and to present treatment schedule proposed by the authors.

## Materials and methods

112 patients with submandibular gland sialadenitis were treated in tertiary university centre (Otolaryngology, Head and Neck Surgery Department Poznań Medical University) in the years 2004–2012. Two time periods were compared between 2004–2008 and 2009–2012. The turning point was December 2008, when sialendoscopy procedure was introduced. 48 patients were treated in the years 2004–2008. Data were analyzed retrospectively on the basis of medical documentation (outpatient charts, operating protocols). The second cohort consisting of 64 consecutive patients was collected prospectively. The epidemiological data (gender, age), duration of complaints, the treatment method and its effectiveness were analyzed and compared in both groups.

In preoperative diagnosis, routine real-time B-mode ultrasonography was applied in all patients. Additionally, in the recent 3 years, CT was performed in 11 cases. During first period, patients were treated in Outpatient Department by incision of mucosa of floor of the mouth in local anesthesia. Following the introduction of sialendoscopy, patients were admitted to the hospital for 1 day. During interventional sialendoscopy, 1.3 and 1.6 mm diameter endoscope (Karl Storz Tutlingen, Germany) was used. Stones were removed with the help of the basket and forceps, introduced through the working canal. The SE procedure was carried out under local anesthesia after premedication with (Midazolam, 7.5 mg). Once the size of the stone localized in submandibular hilum was determined to be larger than 6–7 mm and endoscopic removal was deemed impossible, the decision of combined approach was made. The combined approach (incision of the floor of the mouth at the level of submandibular hilum and sialendoscopy) was performed also under local anesthesia. In case of failure of this treatment and when stone was primarily localized in gland parenchyma, the decision of ESWL or total removal of the gland was undertaken. The open surgery was performed under general anesthesia with the help of facial nerve monitoring, with leads from area of marginal branch around the mouth. Our research was approved by Bioethical Commission. Although the paper had a predominantly descriptive character, some statistical analysis was performed using Spearman and Kruskal–Wallis tests.

## Results

There were 48 patients with submandibular sialolithiasis: 31 outpatient duct incisions with stone evacuation and 17 surgical excision of submandibular gland in the first 5 year period of time. There were 21 men and 27 women, aged from 20 to 73, mean 43. The length of complaints ranged from 3 months to 20 years, mean being 3.4 years. The number of treated patient (in whom the removal of submandibular gland was undertaken) in those years was as follows: 2004–1 patient, 2005–2, 2006–3, 2007–4, 2008–2, 2009–4, 2010–0, 2011–1. In the second, 3.5-year period 158 sialendoscopies were performed, 97 in submandibular glands. Wharton duct stenosis was diagnosed in 33 patients; 64 out of 97 patients with submandibular sialadenitis suffered from lithiasis. There were 38 men and 26 women, aged from 14 to 68, mean 45. The length of complaints ranged from 2 months to 17 years, median 2.9 years.

The first line therapy was sialendoscopy, performed in all patients. Endoscopic removal of stones was possible in 40 cases (62.5 %). In 37 patients, treatment was successful after one endoscopy, 3 patients needed two or more procedures. Double approach (incision of the floor of the mouth in hilar area and sialendoscopy) in patients with refracted, big stones and long history of lithiasis was performed successfully in 21 out of 64 (32.8 %) patients. Surgical excision of the submandibular gland was performed in 3 out of 64 (4.7 %) patients between 2009 and 12. Follow-up in this group of patients ranged from 48 to 6 months, mean being 19.6 months. Comorbidities and patients age had no correlation with sialendosoppy stone removal rates; these were, however, found to be statistically dependent from the duration of complaints and a history of more than 5 years doubled the risk of failure (*p* < 0.005). Various methods of treatment through two distinct time intervals are depicted in Table 1.

**Table 1:** Various methods of treatment throughout two time intervals

Submandibular gland calculi	Years 2004–2008	Years 2008–2012
Removal of calculi through incision of mucous membrane of floor of the mouth in hilar area	31	0
Sialoendoscopy	0	64
Endoscopic removal of calculi	0	40 (62.5 %)
Double approach	0	21 (32.8 %)
Surgical removal of submandibular gland	17	3
Total	48	64

## Discussion

According to Bigler [1], Harrison [4], Yoel [26], Work [25], Wang et al. [24], there are two particular subdivisions: chronic obstructive (sialolithiasis, stenosis of the duct, inflammation of the glandular tissue with recurrent stenosis or enlargement of the duct) and non-obstructive group of inflammations.

Diagnosis of sialolithiasis is based on its clinical presentation and symptoms. Painful, rapidly increasing salivary colic character is exhibited especially during meal time. This agonizing experience may even occur without any component of mechanical obstruction, although presence of lithiasis is the main cause in 50 % of affected individuals. High-resolution ultrasonography could be utilized as an optional diagnostic method for visualization of calcified deposits or exclusion of tumor presence [28]. The main feature of sialography is excision, constriction or enlargement of the excretory ducts. It is not utilized during acute states. Ultrasonographic confirmation of either ductal stricture or presence of inraparenchymal stones allows postponing the sialoendoscopy procedure until the acute state has subsided [6, 8, 13]. High-resolution computer tomography is still considered to be the most sensitive method for the determination of stones, whereas the ultrasonographic technique allows to view a sialolith >2 mm. Increased accumulation of inorganic calcified deposits within the center of the gland gives off a strong echo signal; therefore, it is not uncommon to overlook miniscule remnants of the stones due to insufficient signal saturation. However, it is important to emphasize that false positive results could be obtained in case of excessive hyperemia caused by inflammation of the duct. In these particular situations, sialendoscopy is considered superior. Classic sialography, sialography performed by utilization of computer tomography or by magnetic resonance imaging, is an instrumental addition to the diagnostic evaluation; however, many authors prefer high-resolution ultrasonography [28].

The use of endoscopic and minimally invasive techniques allows for the greater preservation of the major salivary glands in cases of sialolithiasis. According to literature data, 80–90 % of patients with parotid gland sialolithiasis can be treated using minimally invasive techniques such as sialendoscopy and ESWL [5, 6, 8, 9, 14, 15, 17, 19, 27]. It should be remembered that stones larger than 6 mm in diameter and impacted in the wall of the duct limit the possibility of using sialendoscopy [8, 9, 14, 15, 19]. After performing ESWL, larger stones (larger than 8–10 mm in diameter) can successfully be fragmented and then removed using a sialendoscopy. Our initial, 5-year emergency department data indicate that 48 patients who were admitted with the symptoms of sialolithiasis. In 31 patients, stones were removed through an incision in the mucous membrane. Due to complications and persistent symptoms, the remaining 17 patients had their submandibular glands removed. Following the introduction of sialendoscopy procedure to our department, 3.5 years worth of clinical data show 64 patients admitted with symptoms indicating submandibular sialolithiasis. 40 patients (62.5 %) had their stones removed endoscopically. Double approach (incision of the floor of the mouth in hilar area and sialendoscopy) with the successful removal of stones was carried out in 21 out of 64 (32.8 %) patients. Three patients, however, had their submandibular glands totally removed due to several complications.

**Fig. 1:**
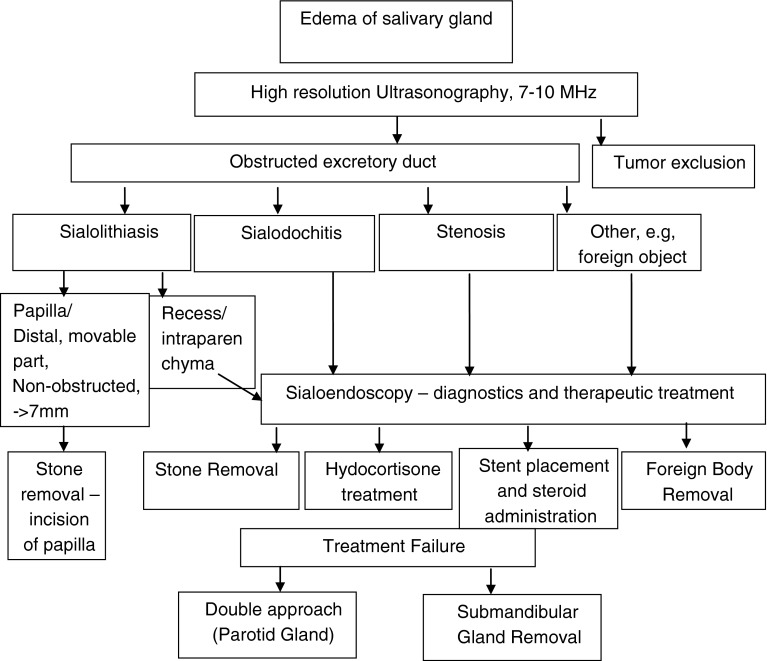
Algorithm of salivary gland obstructive pathology treatment, according to Koch et al

It is valuable to include a diagnostic and therapeutic procedure developed by Koch [11] (Fig. 1). Sialendoscopy is considered as a significant diagnostic and therapeutic method of primary treatment. According to our data, 48 patients treated throughout the 5 year time interval without utilization of sialendoscopy, when compared with those 64 patients who were treated with sialoendoscopy procedure, showed mediocre outcomes. It is, therefore, imperative to point out that the introduction of sialendoscopy and superior results obtained throughout its treatment of chronic inflammation of glands affected by sialolithiasis allowed us to gradually adapt to the published literature [6, 11, 13, 17, 19, 20]. In situation in which there is a limited access to ESWL, a double approach could be used as an alternative method of treatment in both parotid and submandibular glands [10, 13, 16, 18, 23].

The most conservative method of treatment, as well as the one that provides us with the least amount of unfavorable outcomes, is the main objective for those affected by a chronic inflammatory process. According to the literature and our current data, in the vast majority of cases, surgical intervention could be replaced with a minimally invasive procedure by utilization of diagnostic and therapeutic sialendoscopy procedures [6, 11, 13, 19]. According to data obtained at our center, conservative approach was the predominant method of treatment up until the year 2008. The development of sialendoscopy, however, allowed us to treat our patients with a minimally invasive procedures. Similarly, in the year 2004 through 2008, 17 surgical removals of submandibular glands were performed due to lithiasis or advanced inflammatory states; however, in the year 2009–2012, only three were performed.

With the advent of new diagnostic and therapeutic methods, it is imperative to verify current classification of chronic inflammation of large salivary glands. Critical analysis of literature reviews and our own data indicate that continuous improvement of current methods and introduction of new ones, such as utilization of sialendoscopy are crucial in treatment of pathological obstructions of salivary glands. Sialendoscopy is the current treatment of choice for submandibular glands affected by sialoliths. Indication for a complete removal of the gland is becoming uncommon as a first line treatment although still indispensable in chosen cases.
